# 5-year-old child with late discovered traumatic patellar tendon rupture—a case report

**DOI:** 10.1080/17453674.2018.1467846

**Published:** 2018-05-03

**Authors:** Jesper Holbeck-Brendel, Ole Rahbek

**Affiliations:** a Department of Orthopaedic Surgery, Aarhus University Hospital;; b Orthopaedic Research Laboratory, Department of Orthopaedic Surgery, Aarhus University Hospital, Aarhus, Denmark

A 5-year-old otherwise healthy boy was referred to our outpatient clinic with a high-riding patella in the left knee. The patient’s mother had 2 months earlier discovered a small round lump on the patient’s left thigh proximal to where the patella should be. The boy had complained about pain in both legs and the mother discovered the lump during comforting. The boy had had a traumatic incident 5 months earlier, when he fell down from a climbing frame and landed on a flexed left knee. A small discoloration was briefly observed around the knee. The parents noticed no dysfunction and the boy did not complain of pain. He recovered quickly and therefore no further actions were taken.

The boy had no history or clinical signs of collagen disease such as Ehlers-Danlos or osteogenesis imperfecta. He walked normally. Inspection of the left knee revealed a patella alta. There was no effusion or other signs of injury. The boy could extend the left knee against force, but the strength was markedly reduced compared with the right side. He could elevate his left leg with the knee fully extended. The range of motion was normal. Palpation of the left knee joint showed that the patella sat high but it was possible to manipulate it distally to near normal position (–2 cm) compared with the right knee. The ligamentum patellae could not be palpated. The boy did not complain of pain during the examination.

Radiograph of the left knee joint showed marked patella alta (Figure 1). MRI showed signs of injury of the patellar tendon (Figure 2). The MRI was suboptimal due to movement artefacts. Dynamic ultrasound imaging showed a thin, elongated ligamentum patellae, which could not be clearly identified either distally or proximally. There was minimal atrophy of the quadriceps muscle without fatty degeneration. The quadriceps tendon was intact. Furthermore, there were signs of intact medial and lateral patella retinacula.

**Figure 1.**
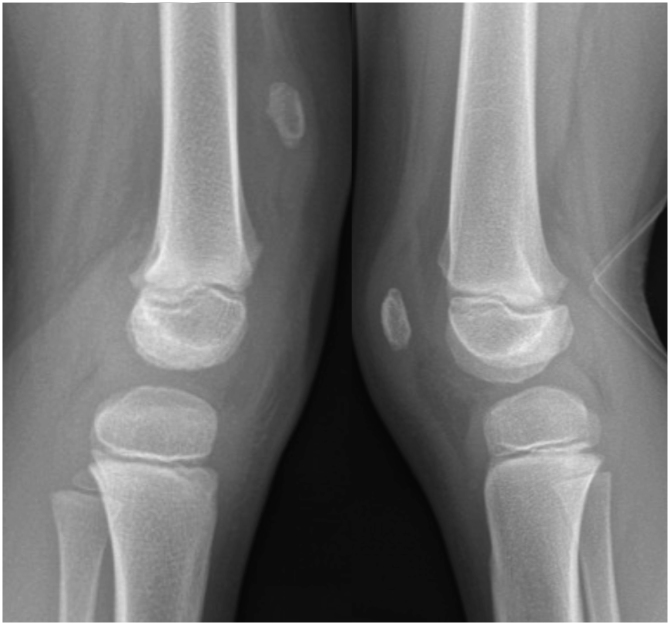
The left and right knee prior to operation.

**Figure 2.**
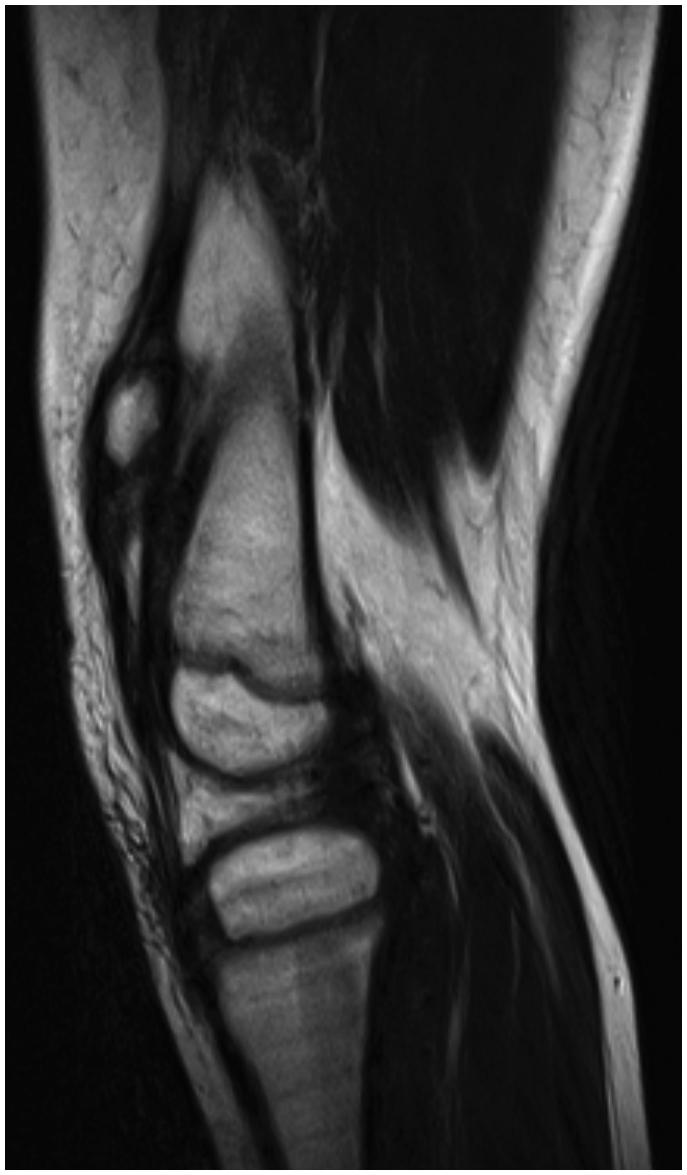
T2 weighted MRI image of the left knee. The patella is proximally located. The ligamentum patella is seen with normal thickness and signal intensity in the most proximal part, but is ruptured distally.

Surgery with repair of the patellar tendon was proposed due to the reduced extension force. It was performed under general anesthesia. The patellar tendon was elongated and shredded, particularly in the distal part were a complete rupture was seen. The proximal two-thirds of the tendon was macroscopically normal and was surgically exposed. The distal part was resected. The lateral and medial retinacula were intact. The periosteum on the tibial tuberosity was surgically exposed and 2 periosteal flaps were made. The patella could be repositioned to a normal position compared with the right side. 2 5.0 Mitek anchors (DePuy Synthes Sports Medicine (Mitek), Raynham, MA, USA) with screw threads were placed in the proximal epiphysis of the tibia with fluoroscopic guidance. The tendon was pulled down and sutured with Vicryl 0 (Ethicon Inc., Somerville, NJ, UA) to the Mitek anchors with appropriate tension compared with the right side. We confirmed the correct placement of the patella central in the femoral groove with fluoroscopic guidance. The strength of the repair was tested by passive knee flexion, which was possible up to 30 degrees when tension increased. A DonJoy brace (DJO Global, Vista, CA, USA) was placed allowing 0–20 degrees of flexion to protect against hyperflexion trauma. Mobilization with full loading on the operated leg was allowed immediately after surgery, but the boy was instructed to refrain from other physical activities.

The boy did not complain of pain during the postoperative period. The DonJoy brace was set to allow 0–80 degrees flexion after 6 weeks. The boy could fully extend the operated knee. Radiographs obtained at 10 weeks postoperatively showed that the patella was positioned just proximal to the physis of the distal femur and still more proximal compared with the right side. The anchors were in place. The boy had no physical limitations and no complaints 1½ years after the operation. Full flexion and extension of the knee joint was possible. The quadriceps strength was normalized. The patella was located 1.5–2 cm proximal on the operated side compared with the right side and moved normally into the femoral groove at full flexion. Radiographs indicated that the patella was in the same position as it was 10 weeks after surgery (Figure 3).

**Figure 3.**
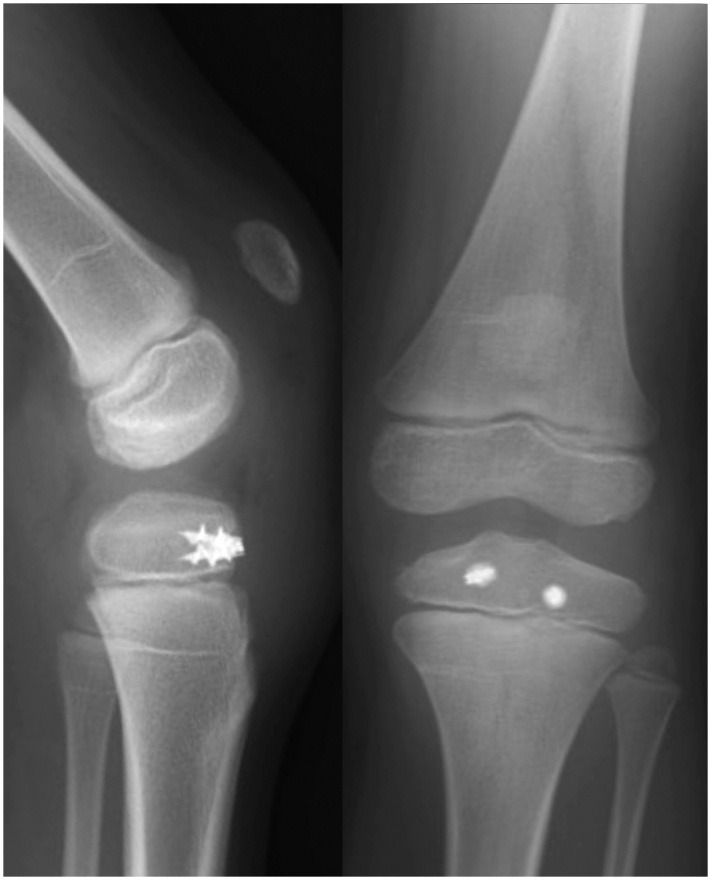
The left knee 1½ years after the operation.

## Discussion

Traumatic rupture of the patellar tendon is extremely rare in preadolescent children (Felt and Arora 2017). It represents 0.6% of the musculoskeletal tendinous injuries in the general population with peak age incidence around 50 years for males and 70 for females (Clayton and Court-Brown 2008).

Considerable force equivalent to 18 times body weight is required to rupture a human adult patellar tendon and as such is very rare in childhood (Pires e Albuquerque et al. 2015). Ruptures of the tendons in children or adolescents are rare; fractures are more common (Williams et al. 2015, Yousef and Rosenfeld 2017). This usually occurs distal to the patellar tendon by an avulsion fracture of the tibial tuberosity or proximally as a patellar sleeve fracture (Berg 1995). Optimal treatment of acute isolated patellar tendon rupture is prompt surgical repair.

Our 5-year-old, otherwise healthy boy functioned normally with a completely ruptured patellar tendon in the left knee joint. The boy did not present the typical signs of traumatic patellar tendon rupture besides a small discoloration around the knee joint shortly after injury, patella alta and markedly reduced left knee extension strength compared with the right side. It is remarkable that the boy could function so well in the period after the injury. We believe that the intact lateral and medial retinacula may have helped with function.

To our knowledge this is the first case with a traumatic patellar tendon rupture at such a young age and furthermore with the injury being 7 months old before diagnosis. We think that a traumatic lesion is the only plausible explanation as no trochlear dysplasia or fatty atrophy of the quadriceps was present, arguing against a congenital or long-lasting abnormality. In addition, patella alta was first noted by the parents after a relevant trauma and not before. Lastly the boy did not have clinical signs of collagen disease and there was no history of this in the family. Thus a pathological tendon elongation seems unlikely (ElGuindy et al. 2011).

Muratli et al. (2005) present a remotely similar case but their patient had bilateral traumatic patellar tendon rupture with the right side being of 1 month’s duration, and the patient was 9 years old. The patient showed patella alta, a depression between the patella and tibial tubercle, and unlike our case marked atrophy of the quadriceps muscle and no ability to actively extend the injured knee.

We suspect that the boy’s patella alta, unchanged during the 6.5-year follow-up, was due to stretching of the ligament and one could argue that augmentation of the patellar tendon rupture with tendon allograft could have prevented this. Furthermore, it could be argued that the repair was fixed too proximally and therefore placed the patella too proximally. However, knee motion and muscle strength became normal.

JHB did the research and wrote the manuscript. OR treated the patient and revised the manuscript.

Informed consent for publication of this case study was obtained from the boy’s parents.

*Acta* thanks Johannes Mayr and Yrjänä Nietosvaara for help with peer review of this study.
